# Air Entrapment in a Pacemaker Pocket in a Child

**DOI:** 10.3390/children13010018

**Published:** 2025-12-22

**Authors:** Vitaliy V. Suvorov, Dmitri O. Ivanov

**Affiliations:** Department of Surgical Diseases of Children, Saint-Petersburg State Pediatric Medical University, 194100 Saint Petersburg, Russia; spb@gpmu.org

**Keywords:** complication, pacemaker, pacemaker pocket air, pacemaker in child

## Abstract

**Highlights:**

**What are the main findings?**

- Implantation of a pacemaker in children is associated with a risk of air entrapment in the pacemaker pocket.
- This complication increases the risk of pacemaker malfunction.

**What are the implications of the main findings?**

- Pacemaker implantation in children should be serially followed by chest radiographs.
- It is important to be aware of this potential unusual complication when considering precautions, diagnostic measures, and treatment options.

**Abstract:**

Background: Advances in pediatric electrophysiology have revolutionized cardiac care by offering patients treatments for increasingly complex cardiac rhythm and conduction disorders. However, despite these innovations, there are a number of potential complications that clinicians have to deal with. Case presentation: This clinical case study describes a rare complication in a child following pacemaker implantation, namely the appearance and accumulation of air in the pacemaker pocket. The child underwent multiple cardiac surgery for a complex congenital heart defect (CHD). Unfortunately, during surgical repair of a ventricular septal defect, the conduction pathways were disturbed. This caused second-degree atrioventricular block and required implantation of an epicardial pacemaker system. Heart block developed several days postoperatively and the child underwent a series of diagnostic tests and was successfully treated. Discussion: Pneumothorax and pneumomediastinum with air dissemination into the pacemaker pocket may develop postoperatively. But this a rare complication of pacemaker implantation, especially in children. This complication can cause pacemaker malfunction and be life-threatening. In the presented clinical case, the most likely cause was spontaneous pneumothorax expanding to the mediastinum and into the pacemaker pocket. Conclusions: Early identification of this complication will minimize the risk of pacemaker dysfunction and improve clinical outcomes.

## 1. Introduction

Over the past two decades, pacemaker technology has evolved significantly, moving from simple single-chamber devices to complex multi-chamber systems for adults and children [1,2]. However, permanent pacemaker implantation has a number of disadvantages and complications, including both mechanical and clinical issues. These not only impact patient outcomes but also pose significant economic challenges. Each complication represents a unique problem requiring an individual approach to treatment [3,4,5].

We describe a case of air accumulation in the pacemaker generator bed, likely resulting from the development of pneumothorax with pneumopericardium and pneumomediastinum several days after implantation of a dual-chamber epicardial pacemaker system in a 9-month-old child.

## 2. Clinical Case

A 9-month-old male infant was admitted to the Pediatric Department of the St. Petersburg State Pediatric Medical University Clinic with a history of surgically corrected congenital heart disease (CHD), including double outlet right ventricle, coarctation of the aorta, hypoplasia of the aortic arch, and two ventricular septal defects (VSDs). The VSDs were an unrestrictive subaortic defect and a restrictive mid-muscular defect. In the neonatal period, at 14 days of age, he had resection of the coarctation with aortic arch reconstruction using a homograft patch and pulmonary artery banding.

At 9 months of age, the child had both VSDs closed with a xenopericardial patch and pulmonary artery repair with an autologous pericardial patch. In the early postoperative period, the child developed hemodynamically significant second-degree atrioventricular block (Mobitz type 2), which required temporary dual-chamber pacing. The heart block persisted during the 13-day postoperative follow-up period.

Holter monitoring demonstrated sinus rhythm with an atrial rate of 150/min, ventricular rate of 75–150/min with episodes of the 2nd-degree Mobitz-II block with a conduction periodicity of 3:2, 4:3. With the temporary pacemaker in place, transthoracic echocardiography showed satisfactory contractile function of the heart and a left ventricular ejection fraction by Simpson of 66%. There was mild mitral valve insufficiency and moderate tricuspid valve insufficiency. The calculated right ventricular systolic pressure was 44 mmHg. A 2 mm atrial septal defect with a small left-to-right shunt was visualized. There was moderate residual right ventricular outflow tract stenosis with maximum instantaneous gradient = 41 mmHg, mean gradient 20 mmHg. The aortic arch had no signs of obstruction. Filling pressures based on inferior vena cava collapse were normal. There was no pericardial or pleural effusion.

Based on these data and the clinical presentation, a permanent epicardial dual-chamber pacing system (Medtronic Attesta ATDR01; mode: DDD; atrial and ventricle leads pace and sense polarity: bipolar; lower rate: 90 ppm; upper track: 160 ppm; paced AV delay: 200 ms) was implanted. Implantation of the epicardial pacing system was performed through a median sternotomy. Because this was a sternal reentry, the pleural cavities, retrosternal space and pericardium all communicated. After cardiac mobilization, electrodes were attached to the atria and ventricles; the electrode impedance, sensitivity, and pacing threshold were measured in selected epicardial zones. A pacemaker pocket was created in the anterior abdominal wall beneath the abdominal muscles. Although it was isolated from the pericardium by suturing, there was still communication with the pericardial space along the electrodes. Immediately postoperative, pacemaker function was normal. Signs of gastrointestinal (GI) infection were noticed in the early postoperative period, accompanied by episodes of fever, loose stools, increased intestinal gas, and abdominal distension. Several attempts to extubate the patient failed, which caused prolonged mechanical ventilation.

**Figure 1:**
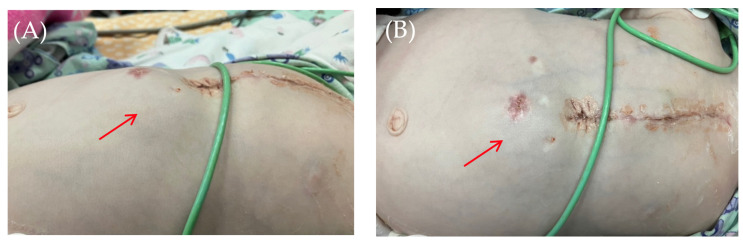
Thoracoabdominal view. Lateral (**A**) and frontal (**B**) views. A mass and soft tissue bulge are noted (red arrow) in the area of the pacemaker pocket in the subxiphoid region of the anterior abdominal wall, limited by the projection of the pacemaker body.

On the 8th postoperative day, the wound healed by primary intention. During pacing programming, impedance values, atrial and ventricular pacing thresholds were satisfactory; no pacing abnormalities were detected. On the 10th postoperative day, local examination in the upper anterior abdominal wall along the midline revealed a localized bulge of skin and soft tissue in the projection of the pacemaker pocket (Figure 1).

**Figure 2:**
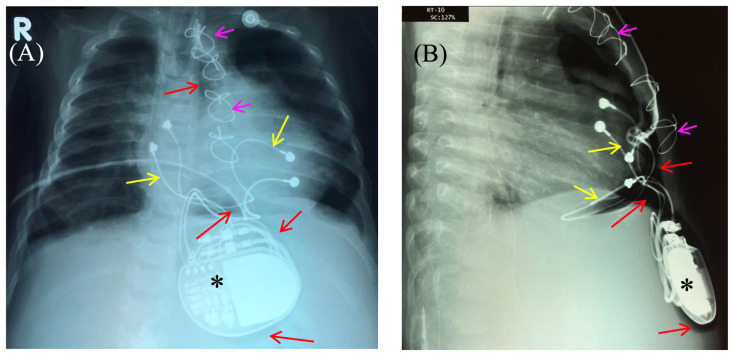
Chest X-ray—frontal (**A**) and lateral (**B**) view. Chest X-ray shows no pneumothorax, but pneumopericardium, pneumomediastinum and air in the pacemaker pocket site (red arrow) are present. One can also see the pacemaker electrodes (yellow arrow), the pulse generator (black asterisk), and the wire ligature (pink arrow). R: right side.

**Figure 3:**
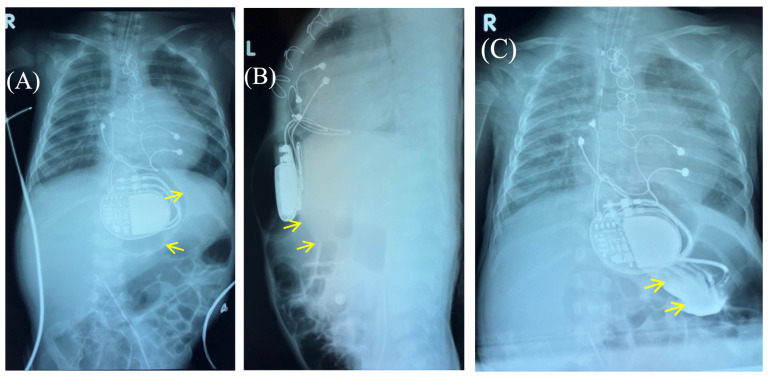
Control of the passage of radiocontrast agent (yellow arrow) through the gastrointestinal tract to exclude perforation of a hollow organ of the abdominal cavity. Frontal (**A**,**C**) and lateral (**B**) view of the chest X-ray 1 h (**A**,**B**) and 2.5 h (**C**) after radiocontrast agent administration. R: right side; L: left side.

The skin in the area of the abdominal wall soft tissue bulge had normal color and temperature. Palpation revealed soft tissue with no detectable fluctuations. The pacemaker pocket was palpated within its bed. To rule out pneumothorax and perforation of a hollow abdominal organ, chest and abdominal radiography and fluoroscopy were performed, as well as GI contrast imaging (Figure 2 and Figure 3).

**Figure 4:**
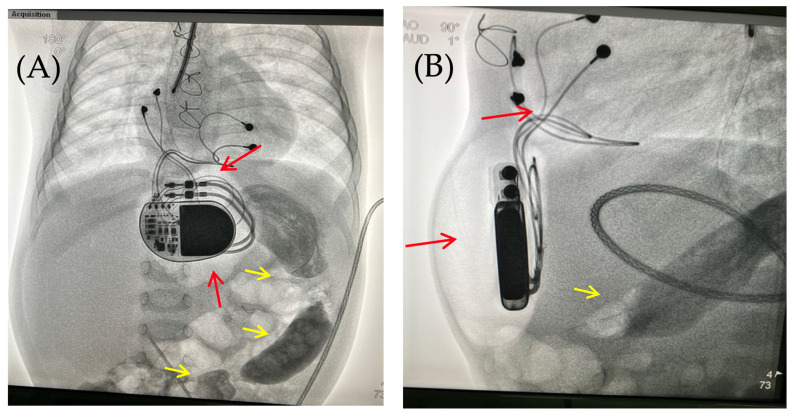
Chest X-ray examination—frontal (**A**) and lateral (**B**) view. Chest X-ray shows no pneumothorax, but pneumopericardium, pneumomediastinum with air are present at the pacemaker pocket site (red arrow). Radiocontrast agent (yellow arrow) at the gastrointestinal tract.

Fluoroscopy and chest and abdominal radiography revealed no signs of pneumothorax. A demarcated area of free air was visualized in the upper anterior abdominal wall along the midline, projecting to the pacemaker pocket. The passage of radiocontrast agent through the gastrointestinal tract was observed dynamically and found to be normal. No signs of hollow organ perforation or intestinal obstruction were detected (Figure 2, Figure 3 and Figure 4).

Radiographic evidence for air in the pacemaker pocket with concomitant gastrointestinal infection raised the high probability of an anaerobic infection. Due to suspicion for surgical infection. The patient returned to the operating room for the revision and debridement of the wound in the area of the pacemaker pocket with bacterial cultures of the wound discharge.

**Figure 5:**
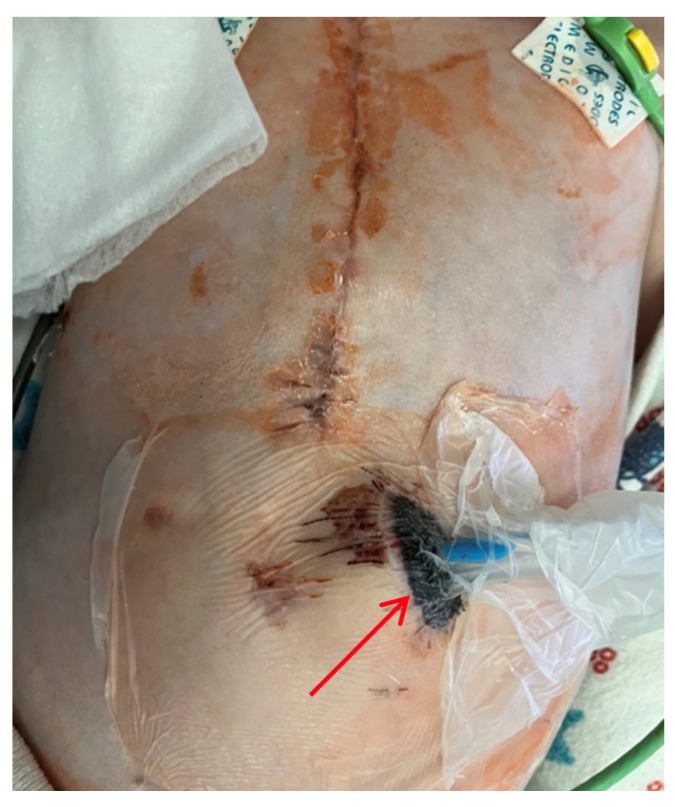
Thoracoabdominal view after wound exploration and pacemaker pocket. Frontal view. A dressing with drainage (red arrow) has been placed in the subxiphoid region of the anterior abdominal wall to perform vacuum drainage.

Intraoperatively, following antiseptic treatment and prior to skin incision, a puncture was performed in the cavity of the pacemaker pocket. The air was evacuated and there was no discharge. Five ml of isotonic saline was administered, the cavity was evacuated, and wound cultures were taken. This was followed by a 5 cm oblique skin incision on the left lateral aspect of the anterior abdominal wall in the subcostal region. The soft tissues were bluntly dissected. The pacemaker pocket was opened. A wound culture was taken. At the revision, the soft tissues were pink and bleeding, with no signs of wound infection. The wound was irrigated with antiseptic solutions. A vacuum drainage system was used (Figure 5).

**Figure 6:**
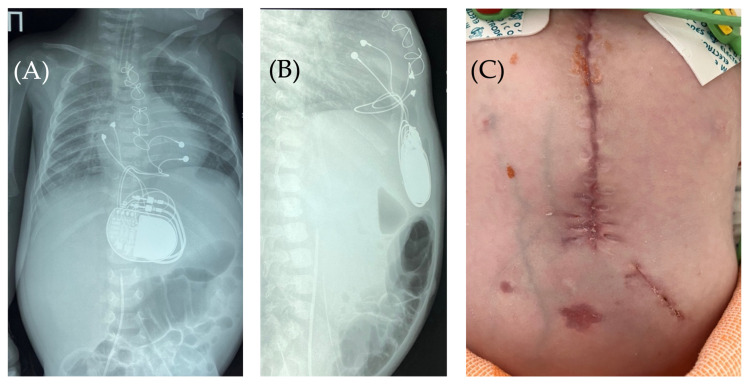
Chest X-ray after surgery debridement and closure of the wound. Chest X-ray (frontal (**A**) and lateral (**B**) view) shows no signs of pneumothorax, pneumopericardium, pneumomediastinum with minimal air at the pacemaker pocket site. (**C**) The healed sternotomy and the projection of the pacemaker pocket 12 days after revision and sanitation.

Based on the results of three wound cultures, no pathogenic flora were detected. On the seventh day, the wound was sutured, and healing proceeded without complications. The wound healed seven days after suture closure. A follow-up chest and abdominal X-ray revealed no signs of pneumothorax, pneumomediastinum, or free air in the upper anterior abdominal wall along the midline in the projection of the pacemaker pocket (Figure 6).

## 3. Discussion

Complications associated with permanent pacemaker implantation procedure usually occur in the early postoperative period. These complications are classified based on the timing of implantation (acute or chronic), location (lead or pacemaker dysfunction, or involvement of the pacemaker bed), and various etiologic causes (not related to the pacemaker system but leading to disruptions in its function or to the deterioration in the patient’s clinical condition). Types of complications may depend on the method of lead implantation—endocardial versus epicardial. The main complications associated with endocardial implantation include: bleeding, electrode displacement and malfunction, pacemaker device migration, venous thrombosis, hemo-/pneumothorax, and perforation of cardiac chambers.

In most young children, an epicardial pacing system is used [6]. Epicardial pacing is associated with complications, primarily related to mechanical stress on the electrodes or a pacemaker: electrode damage, its displacement or detachment from the fixation site, high stimulation threshold, cardiac strangulation, and coronary artery stenosis [6]. Pneumothorax, pneumopericardium or pneumomediastinum are rare complications of epicardial and endocardial pacemaker lead placement [7].

Pneumothorax during pacemaker implantation accounts for no more than 2.5% of cases of endocardial electrode implantation in adults [8] and this complication is more rare in children [4,6]. The development of pneumothorax often requires emergency care in the form of pleural drainage. However, it is also important to note that this complication can contribute to the development of pneumomediastinum, and in the case of pacemaker implantation with epicardial electrode fixation, there is a risk of air entering the pocket.

There are limited published data that air in the generator pocket impairs pacemaker function and contributes to clinical deterioration [7,9,10,11]. Hearne et al., described air accumulation in the area of the pacemaker pocket in an adult patient, leading to pacemaker dysfunction and clinical deterioration [11]. The authors associated the entry of air into the pacemaker pocket with subclavian venipuncture before implantation of the pacemaker electrode. As a result, the anode contact plate of the pacemaker body was isolated from the soft tissues due to air accumulation, and unipolar myocardial stimulation was disrupted.

In a recent study (2025) by Singh and colleagues, an adult with spontaneous asymptomatic pneumothorax also experienced pacing failure in the unipolar mode [9]. The authors noted that the endocardial electrode was placed with passive fixation. The development of the pneumothorax led not only to pulmonary atelectasis but also to mediastinal displacement, resulting in the electrode dislodgement, which contributed to the disruption of myocardial electrical impulse capture. Treatment of the pneumothorax and switching to a bipolar pacing mode successfully resolved this urgent issue.

In our clinical case, the possible mechanism for the development of air in the pacemaker pocket was related to prolonged mechanical ventilation and gastrointestinal infection. Most likely, ventilator-associated lung injury caused the development of a pneumothorax that extended into the anterior mediastinum, forming a pneumomediastinum and pneumopericardium. Due to the communication between the pericardium and the anterior mediastinum, air entered the pacemaker pocket and accumulated there. As previously mentioned, the disruption of contact between the housing of most pacemaker generators with soft tissue can cause fatal pacing malfunctions. However, the type of pacemaker implanted in this case did not require constant contact with the patient’s soft tissues to ensure normal operation. Therefore, no disruptions in pacemaker function or deterioration of the patient’s clinical condition were observed.

## 4. Conclusions

This case highlights the risks of pneumothorax, pneumopericardium, and pneumomediastinum with air dissemination into the pacemaker pocket in children. It is important to be aware of these rare complications, so that proper precautions, diagnostic measures, and treatment options can be undertaken. However, there are currently no guidelines in place regarding pneumothorax or pneumopericardium avoidance during pacing device implantation or regarding air entry into a pacemaker pocket.

## Data Availability

The datasets generated during and/or analyzed during the current study are available from the corresponding author upon reasonable request. The data are not publicly available due to privacy restrictions.
